# Geographic inequalities and gender-specific trends in oropharyngeal cancer mortality in Spain

**DOI:** 10.4317/medoral.27957

**Published:** 2026-01-24

**Authors:** Lucia Cayuela, Julian Librero, Pedro Infante-Cossio, Jose-Juan Pereyra-Rodriguez, Aurelio Cayuela

**Affiliations:** 1 Department of Internal Medicine, Hospital Severo Ochoa, Leganes, Spain; 2 Navarrabiomed-HUN-UPNA, Red de Investigacion en Cronicidad, Atencion Primaria y Prevencion y Promocion de la Salud (RICAPPS), Pamplona, Spain; 3 Department of Oral and Maxillofacial Surgery, Virgen del Rocio University Hospital, University of Seville, Seville, Spain; 4 Department of Dermatology, Virgen del Rocio University Hospital, University of Seville, Seville, Spain; 5 Department of Medicine. School of Medicine, University of Seville, Seville, Spain; 6 Independent researcher, Seville, Spain

## Abstract

**Background:**

Consistent geographical disparities in oropharyngeal cancer (OPC) mortality rates have been observed across Europe, with distinct trends emerging among men and women. This study provides a detailed subnational analysis of OPC mortality in Spain between 1999 and 2023, categorised by gender and administrative region.

**Material and Methods:**

We conducted an ecological analysis using ICD-10 C10 mortality data for all 50 Spanish provinces and the two autonomous cities (Ceuta and Melilla). Hierarchical Bayesian spatio-temporal Poisson models, fitted via INLA, were employed to decompose variance and identify gender-specific risk patterns.

**Results:**

Gender-specific patterns were markedly distinct. Male mortality showed a strong spatial structure (88.1% of variance), with persistent high-risk clusters in Northern Spain (Asturias, A Coruña, Cantabria) and the Canary Islands. Despite a modest national decline, risk often remained stable or increased in these high-risk areas; for instance, Cantabria's relative risk (RR) peaked at 2.62 in 2023. This pattern is consistent with entrenched traditional risk factors (tobacco and alcohol use). Conversely, female mortality was dominated by a strong national temporal increase (17.2% of variance), likely reflecting a growing HPV-associated burden. Risk escalated nearly uniformly across the country, rising from RR 0.62 in 1999 to 1.54 in 2023. Localised temporal deviations were significant for men (10.2% interaction), but negligible for women (0.4%), confirming a synchronised, nationwide female trend.

**Conclusions:**

OPC mortality dynamics demand dual intervention strategies. Deep-rooted gender disparities in the northern and island provinces suggest strengthening tobacco and alcohol control campaigns. The rapid and widespread increase in female mortality highlights the need of expanding HPV vaccination programmes nationwide.

## Introduction

Oropharyngeal cancer (OPC) poses an escalating public health challenge, characterised by substantial geographical disparities in mortality across Europe (1). While male OPC mortality has declined in several Southern European countries, rates have plateaued or risen in Eastern Europe and the United Kingdom, reflecting persistent inequalities in prevention, diagnosis, and care (1 , 2). By contrast, female mortality has remained stable or increased in many settings, signalling a shifting risk profile largely driven by human papillomavirus (HPV)-related diseases (1 - 3). These divergent trends mirror global patterns, in which reductions in tobacco-related and alcohol-related cancers coexist with an increase in HPV16-associated OPC, particularly in high-income countries (1 , 3 - 5).

National data from Spain reveal a similarly complex epidemiological landscape. Although oral cavity cancer (OCC) mortality has declined, OPC mortality has stabilised or increased slightly, especially among middle-aged adults (6 - 8). The prevalence of HPV-related OPC is rising, paralleling the trajectories observed in Northern Europe and North America (3). Projections are concerning: Despite an anticipated overall decline in OPC mortality -from 1.23 per 100,000 in 2015-19 to 0.71 per 100,000 in 2040-44-rates among women aged 65 years and older are expected to rise (9). This pattern aligns with emerging evidence that HPV-related OPC increasingly affects older adults of both genders, potentially reflecting evolving sexual behaviours and cumulative lifetime HPV exposure (1 , 3).

Substantial subnational disparities exist within Spain. Northern regions, particularly the Basque Country, consistently exhibit the highest OPC incidence and mortality, indicating a pronounced geographical gradient (3 , 8 , 10 - 12). These variations are probably linked to historical differences in tobacco and alcohol consumption, socioeconomic inequalities, and differential access to early diagnosis and specialised care, with the strongest effects among men and older populations. Although the overall prevalence of HPV-related OPC in Spain remains lower than in Northern Europe and North America, its steady increase suggests an ongoing epidemiological transition (3 , 11). Understanding the spatio-temporal evolution of these disparities is crucial to inform targeted prevention strategies, optimise resource allocation, and reduce inequities in outcomes.

We applied hierarchical Bayesian spatio-temporal models to OPC mortality data across Spain's 50 provinces and two autonomous cities from 1999 to 2023, stratified by gender and year, to characterise these evolving geographical and temporal patterns.

## Material and Methods

This province-level ecological study examined long-term trends in OPC mortality in Spain between 1999 and 2023. Mortality was classified using ICD-10 code C10 (malignant neoplasm of the oropharynx), selected to ensure consistency with contemporary epidemiological research, including global assessments of HPV-related cancer burden, and to maximise comparability across studies (1). The analysis included all 50 provinces and the two autonomous cities of Ceuta and Melilla.

Mortality and population data were obtained from the National Institute of Statistics (http://www.ine.es), disaggregated by province, gender, calendar year, and five-year age group (0-4 to85 years). These data supported robust age standardisation. Expected deaths were derived using indirect standardisation by applying national age-specific OPC mortality rates to the corresponding provincial and administrative region populations. For descriptive purposes, crude mortality rates (CMR) and standardised mortality ratios (SMR), calculated as the ratio of observed to expected deaths, were determined and are presented in Table 1. However, to obtain more stable and precise small-area estimates, the main analyses relied on hierarchical Bayesian spatio-temporal modelling.

Table 1

Spatial relationships between provinces were defined using a graph-based neighbourhood structure based primarily on Queen contiguity. For geographically isolated provinces, a k-nearest neighbours approach was incorporated to ensure a fully connected spatial graph, a prerequisite for stable estimation of spatial random effects.

Smoothed relative risks (RRs) were estimated using a Bayesian spatio-temporal Poisson log-linear mixed model of the form Oit - Poisson(EitRRit), log(RRit)= + ui + vi + t + it, where Oit and Eitt are observed and expected deaths for province i in year t; is the overall intercept; ui and vi denote spatially structured and unstructured random effects; t represents the temporal component; and it captures the spatio-temporal interaction.

Four alternative spatial structures were evaluated: The intrinsic Conditional Autoregressive (iCAR) model, the Besag-York-Mollié (BYM) model, its reparametrized form (BYM2), and the Leroux Conditional Autoregressive (CAR) model. Temporal trends were modelled using first- and second-order random walks (RW1 and RW2). Spatio-temporal interactions were specified following the Knorr-Held typology (Types I-IV) and an additive form, providing flexibility in delineating province-specific temporal deviations. Penalised Complexity (PC) priors were applied to all hyperparameters to encourage parsimony and mitigate overfitting (13).

Models were fitted using Integrated Nested Laplace Approximation (INLA) implemented in the R-INLA package. In total, 160 candidate models were estimated-80 for each gender-arising from combinations of four spatial structures, two temporal formulations, five interaction types, and two PC-prior configurations. Model performance was assessed using the Deviance Information Criterion (DIC) and the Widely Applicable Information Criterion (WAIC), and final model selection was guided by both goodness-of-fit and predictive parsimony.

Posterior estimates of excess mortality were summarised using posterior probabilities (PP) that RRit&gt;1. Mortality risk was classified as low (PP&lt;0.20), moderate (0.20-0.80), or high (PP&gt;0.80), with PP0.95 indicating strong evidence of elevated risk. Spatial and temporal patterns of OPC mortality were visualised using choropleth maps generated in R, depicting the geographical distribution and evolution of risk across Spanish provinces.

## Results

Model selection and variance decomposition

Different optimal Bayesian hierarchical spatio-temporal models were identified for men and women, indicating gender-specific epidemiological processes underlying OPC mortality in Spain from 1999 to 2023. For men, the best-fitting model combined a Leroux conditional autoregressive (CAR) spatial prior, a first-order random walk (RW1) temporal effect, and a Type II spatio-temporal interaction (DIC 5,037.37; WAIC 5,051.31). For women, the optimal model used a Besag-York-Mollié 2 (BYM2) spatial prior, a second-order random walk (RW2) temporal effect, and a Type III interaction (DIC 2,428.70; WAIC 2,426.60).

Variance decomposition supported these structural differences. In men, mortality risk was dominated by stable spatial heterogeneity (88.1% of total variance), with the spatio-temporal interaction contributing 10.2%. In women, mortality was also strongly spatially structured (82.4% of variance; clustering parameter =0.82), but the national temporal trend accounted for a larger share of variability (17.2%). Baseline mortality was slightly lower in women (RR intercept -0.17) than in men (RR intercept -0.12).

Temporal trends

**Figure 1:**
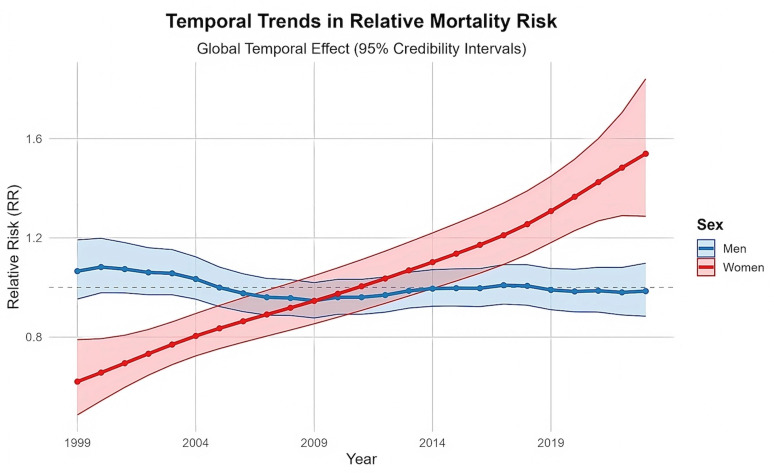
Temporal trends in sex-specific relative risk of OPC mortality in Spain, 1999–2023.

Among men, the national temporal trend (Figure 1) showed a slow and irregular decline. The temporal relative risk (RR) peaked in 2000 (RR 1.082), fell below unity in 2005, and remained marginally below one by 2023 (RR 0.985). The Type II spatio-temporal interaction captured meaningful provincial departures from this pattern; for example, Cantabria reached an RR of 2.62 in 2023, indicating a substantial local increase in risk despite the modest national reduction. These local deviations, which accounted for 10.2% of total variance, underline pronounced temporal heterogeneity across provinces.

1

Female OPC mortality exhibited a contrasting evolution, driven by a strong and coherent national trend (Figure 1). The temporal RR started well below one in 1999 (RR 0.620), increased steadily, crossed unity in 2011 (RR 1.004), became statistically significant from 2014 onwards (PP0.95), and peaked in 2023 (RR 1.539; 95% CrI 1.287-1.841). The minimal contribution of the spatio-temporal interaction (0.4%) indicates that the spatial pattern remained largely stable over time, with all provinces experiencing a broadly synchronised rise in absolute risk.

Spatial patterns

**Figure 2:**
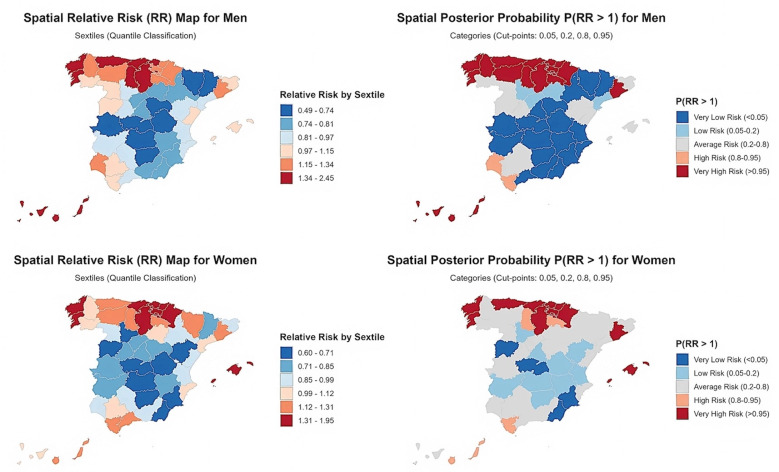
Spatial distribution of OPC mortality risk in Spanish provinces among men and women, 1999–2023.

Male OPC mortality displayed marked and persistent geographical disparities (Figure 2).

2

The highest significantly elevated RRs (PP0.95) were concentrated in Northern Spain and the Canary Islands, notably Las Palmas (RR 2.45) and Santa Cruz de Tenerife (RR 1.34). On the mainland, the principal high-risk belt in the north comprised Asturias (RR 2.18), A Coruña (RR 2.06), Burgos (RR 1.94), Vizcaya (RR 1.81), and Cantabria (RR 1.81). In contrast, an extensive cluster of significantly low RRs (PP0.05) spanned the central and southern interior, the Mediterranean coast, and the Madrid region. The lowest risks were observed in Cuenca (RR 0.486) and Lleida (RR 0.521), followed by Toledo (RR 0.564) and Huesca (RR 0.562); other low-risk provinces included Madrid (RR 0.774), Alicante (RR 0.809), and Valencia (RR 0.839). Provinces such as Sevilla (RR 1.000) and Islas Baleares (RR 0.985) showed a non-significant risk. Overall, these patterns define a pronounced north-west-to-south-east gradient in male OPC mortality, with the Canary Islands forming a distinct insular high-risk zone.

In women, mortality was also strongly clustered spatially (82.4% of variance), but the geography of risk was less extreme and more compact than in men (Figure 2). The highest average spatial RRs formed a discrete high-risk cluster in Northern Spain, led by Navarra (RR 1.95) and Cantabria (RR 1.95), and extending to provinces such as Barcelona (RR 1.28). A smaller low-risk cluster (PP0.05) was identified in the central-western and south-eastern regions, with the lowest risks in Toledo (RR 0.596) and Salamanca (RR 0.606), and significantly low RRs also in Almería (RR 0.649) and Murcia (RR 0.707). Remaining provinces, including A Coruña (RR 1.317) and Asturias (RR 1.296), fell within the non-significant range.

Spatio-temporal dynamics

**Figure 3:**
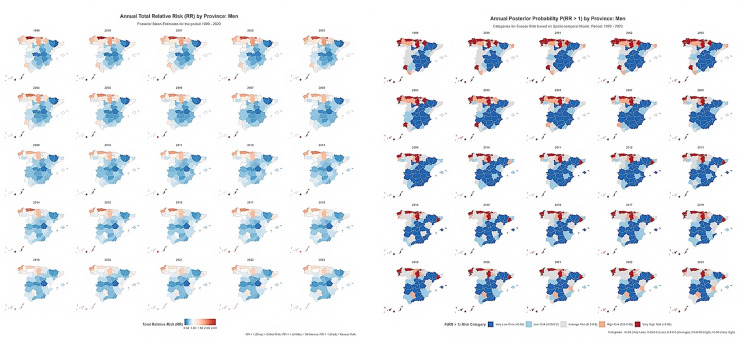
Spatio-temporal trends in OPC mortality risk among men in Spanish provinces, 1999–2023.

**Figure 4:**
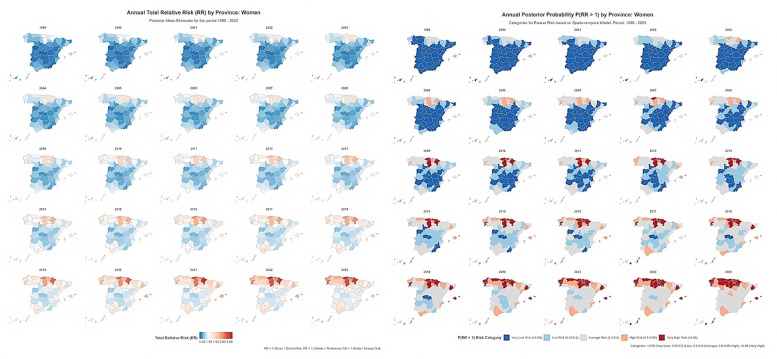
Spatio-temporal trends in OPC mortality risk among women in Spanish provinces, 1999–2023.

Spatio-temporal maps (Figure 3 and Figure 4) showed that the broad geographical gradient in OPC mortality persisted throughout the study period.

3

4

Among men, the northern/insular high-risk cluster remained highly stable, consistently presenting the highest total RRs across all time windows (1999-2003 through 2019-2023), while the central and southern low-risk cluster persisted and supported the modest national decline. Provinces in this low-risk zone-maintained RRs significantly below one, indicating sustained success in limiting mortality. The relatively high interaction variance reflects divergent trajectories in absolute risk: Although the national trend was slightly downward, some provinces experienced localised increases, exemplified by Cantabria (RR 2.62 in 2023), suggesting local drivers that counteracted national gains and preserved the spatial gap.

In women, the negligible spatio-temporal interaction (0.4%) is consistent with the Type III interaction structure, confirming that the pronounced national temporal acceleration was largely synchronous across provinces. Annual maps (Figure 4) reveal a progressive, countrywide shift in risk: In the early 2000s most provinces were characterised by deficit risk (RR&lt;1.0), whereas by the late 2010s the majority displayed excess risk (RR&gt;1.0), with little reorganisation of high- and low-risk areas. The established northern high-risk cluster intensified (higher absolute RRs), while previously low-risk regions transitioned to non-significant or moderate excess risk. This widespread, synchronised increase in absolute mortality risk suggests that the principal determinants of female OPC mortality operate at the national level rather than being confined to specific territories.

## Discussion

This study provides the most comprehensive spatio-temporal characterisation of OPC mortality in Spain to date, identifying enduring geographical inequalities, strong spatial clustering, and pronounced gender-specific patterns over a 25-year period. In men, we observed marked north-south and insular-mainland gradients, particularly elevated risks in Asturias, A Coruña and the Canary Islands, and only modest national declines since the early 2000s. The persistence or intensification of high-risk areas suggests entrenched local determinants. Although female mortality was substantially lower, it exhibited a clearer temporal increase and more homogeneous spatial dependence, consistent with the growing contribution of HPV-related disease in high-income settings (1 , 3).

Our findings align with European studies reporting north-south contrasts and male predominance in OPC mortality (14 , 15). By applying a fully Bayesian spatiotemporal framework implemented via the INLA, we extend previous work by quantifying the relative contributions of spatial, temporal, and interaction components to total variance (13 , 16). In men, spatial structure dominated (88% of total variance), whereas women showed slightly lower spatial dominance (82%), reflecting more pronounced temporal effects (17% versus 2% in men). This aligns with international reports of rising OPC mortality in females and corresponds with shifts in HPV prevalence, as well as evolving tobacco and alcohol consumption patterns (17).

Recent global analyses have consistently demonstrated an increasing incidence of OPC across multiple countries. This rise is primarily driven by HPV-related birth cohort effects, which are now extending into older age groups among men but show more variable patterns in women (17). Crucially, HPV-related OPC typically affects individuals who do not smoke or drink alcohol, primarily targets the tonsils and the base of the tongue, and is often diagnosed at an advanced stage. This stands in sharp contrast to the trends observed for OCC, as demonstrated by the Spanish data spanning 1979 to 2018. While the overall OCC mortality rate in Spain declined during this period, the rate among men initially increased, peaking between 1979 and 1992 before subsequently stabilising (6). This divergence is highly significant: It reflects the successful reduction in OCC traditionally associated with tobacco and alcohol consumption, likely resulting from cultural, social, and legislative changes implemented in the 1980s. These changes led to a measurable decline in the incidence and mortality of cancers linked to traditional risk factors, which is now offset by the concurrent and alarming increase in HPV-associated OPC (3 , 6 , 8 , 11).

High-risk clusters in Northern Spain mirror previously reported head and neck cancer mortality patterns, particularly in Asturias and Galicia, historically characterised by elevated smoking, alcohol consumption, and occupational exposures in mining and industry (18 , 19). The persistent excess in the Canary Islands may reflect unique risk factor profiles, delayed prevention implementation, or unmeasured lifestyle determinants. Conversely, low-risk central provinces such as Cuenca, Toledo, and Ciudad Real coincide with a lower historical tobacco prevalence and higher socioeconomic stability. Comparable patterns have been documented for lung and oesophageal cancer along the Cantabrian coastline, suggesting shared aetiological factors related to tobacco and alcohol consumption (10 , 18).

The persistence of high-risk clusters among men indicates that traditional carcinogenic exposures -mainly tobacco and heavy alcohol consumption-remain influential. This pattern persists despite decades of national campaigns and legislation, likely reflecting the long latency between exposure and cancer manifestation. Thus, current geographical disparities may largely reflect historical, rather than contemporary, high-risk behaviours. Between 2009 and 2020, daily smoking prevalence declined by 8 percentage points in men and 5 in women, with reductions in cigarette intensity (20). These modest national declines are likely the result of cumulative tobacco-control measures, reduced heavy drinking, and improvements in early detection and treatment (20 , 21). Nevertheless, entrenched high-risk clusters suggest that cultural norms, occupational exposures, and socioeconomic inequalities continue to sustain localized risk.

In women, the steeper temporal increase and strong spatial clustering point to a dual dynamic: Changing behavioural exposures across cohorts and the growing volume of OPC cases potentially associated with HPV. HPV now accounts for approximately 70% of OPC incidence in high-income countries, with HPV-16 responsible for 85-90% of cases in North America (22). HPV-positive OPC incidence has increased notably among younger adults and non-smokers, whereas HPV-negative OPC linked to traditional risk factors has declined (17 , 23). Geographic and demographic variations in HPV exposure reflect differences in sexual behaviours and oral HPV prevalence (1 , 24). Our RW2 temporal model captures these gradual but accelerating risk changes over time.

The minimal spatio-temporal interaction in women (0.4%) and moderate effect in men (10%) suggest that most mortality variation is geographically entrenched rather than driven by rapidly changing local trends. Nevertheless, emerging deviations in provinces such as Navarra and Cantabria highlight the need for continued surveillance. Combined tobacco and alcohol use remains highly carcinogenic, with a 30-fold increased risk compared with either substance alone (25). Spain's relatively high alcohol consumption and low excise taxes, particularly on wine, indicate opportunities for targeted public health interventions (26).

This study has two key strengths. First, it spans 25 years (1999-2023), providing sufficient cases to capture major epidemiological transitions and robust temporal trends. Second, the use of small-area Bayesian spatio-temporal models with INLA enabled stable estimation even in sparsely populated provinces, efficiently quantifying uncertainty and overcoming the computational limitations of traditional MCMC methods. The systematic evaluation of multiple spatial, temporal, and interaction structures (160 models) further reinforced the robustness of our findings (27).

Several limitations should be acknowledged. The ecological design and the reliance on mortality data, together with the absence of population-level HPV status, limit causal inference. Important determinants of OPC outcomes -such as HPV seroprevalence and vaccination coverage, gender-specific tobacco and alcohol use, sexual behaviour, and socioeconomic inequalities-could not be accounted for at the provincial level. Variations in healthcare access, tumour characteristics, and diagnostic pathways may also contribute to residual spatial differences. Despite age-standardisation, population ageing and internal migration may have influenced risk patterns. Finally, provincial-level analyses may mask sub-provincial heterogeneity, particularly in large or socioeconomically diverse regions. Future studies employing scalable Bayesian approaches at finer spatial resolutions could help address these limitations (27).

The persistence of high-risk clusters in Northern Spain and the Canary Islands highlights regions where intensified public health action could yield substantial benefit. Targeted policies -including strengthened tobacco and alcohol control, improved screening, and equitable access to specialised oncological care-are strongly warranted. It is unlikely that advances in treatment or overall care quality have markedly influenced long-term mortality, given the modest survival gains observed. Moreover, the stability of the universal public health system in Spain indicates that structural access to healthcare is less likely to explain these geographical disparities (3 , 28).

These trends highlight the urgent need for renewed political commitment to comprehensive control policies, especially in light of limited legislative progress on tobacco over the past 15 years (20). Increasing taxes on tobacco and alcohol remains a high-impact, evidence-based intervention (26 , 29). With HPV vaccination introduced in 2007, its long-term effect on mortality is still uncertain, yet rising mortality in women reinforces the importance of expanded vaccination and enhanced surveillance for HPV-related OPC. Given the higher burden of HPV-positive disease in men, gender-neutral vaccination strategies are strongly recommended (23 , 24).

Future research should integrate incidence, survival, and HPV data and employ multivariate Bayesian models to improve causal inference and guide targeted interventions (30). Although the protective effect of HPV vaccination against OPC is not yet fully established, rising mortality supports educational campaigns on HPV transmission. Continued gender-specific monitoring and prevention of traditional risk factors remain essential.

## Conclusions

OPC mortality in Spain shows persistent geographical disparities and contrasting gender-specific trends. Male mortality is slowly declining overall but remains high in northern and island provinces, while female mortality is rising nationwide, reflecting the growing impact of HPV-related diseases. These findings highlight the need for region-specific interventions, including strengthened tobacco and alcohol control, wider HPV vaccination, and continued surveillance. Bayesian spatio-temporal modelling via INLA provides a robust tool to guide targeted cancer control strategies.

## Figures and Tables

**Table 1:** Table Observed (O) and expected (E) deaths from OPC, crude mortality rates (CMR) and standardized mortality ratio (SMR) in Spanish administrative regions, 1999-2023.

	Men				Women			
	O	E	CMR	SMR	O	E	CMR	SMR
Albacete	40	53.91	0.83	0.74	6	8.53	0.12	0.70
Alicante	188	256.57	0.86	0.73	43	39.74	0.19	1.08
AlmerÃ­a	54	80.27	0.64	0.67	5	12.20	0.06	0.41
Álava	54	47.21	1.39	1.14	11	7.45	0.28	1.48
Asturias	342	172.99	2.72	1.98	37	30.31	0.27	1.22
Ávila	24	28.98	1.15	0.83	4	4.47	0.19	0.89
Badajoz	68	94.97	0.82	0.72	7	15.18	0.08	0.46
Balears, Illes	119	135.88	0.91	0.88	30	21.22	0.23	1.41
Barcelona	885	726.61	1.35	1.22	149	123.61	0.22	1.21
Bizkaia	285	175.43	2.06	1.62	45	30.00	0.31	1.50
Burgos	110	59.34	2.43	1.85	20	9.26	0.45	2.16
CÃ¡ceres	41	63.51	0.82	0.65	9	10.11	0.18	0.89
CÃ¡diz	144	153.54	0.96	0.94	27	24.05	0.18	1.12
Cantabria	140	86.36	2.01	1.62	29	14.56	0.40	1.99
CastellÃ³n	78	78.96	1.11	0.99	11	12.42	0.16	0.89
Ceuta	11	8.89	1.10	1.24	2	1.31	0.21	1.53
Ciudad Real	44	70.79	0.71	0.62	6	11.64	0.09	0.52
CÃ³rdoba	98	106.55	1.02	0.92	17	17.83	0.17	0.95
CoruÃ±a, A	327	175.65	2.43	1.86	37	30.38	0.25	1.22
Cuenca	10	33.08	0.39	0.30	2	5.19	0.08	0.39
Gipuzkoa	128	107.21	1.49	1.19	29	17.83	0.32	1.63
Girona	96	98.53	1.08	0.97	11	15.35	0.12	0.72
Granada	81	118.79	0.74	0.68	16	19.22	0.14	0.83
Guadalajara	17	31.74	0.57	0.54	2	4.77	0.07	0.42
Huelva	70	65.07	1.12	1.08	9	10.34	0.14	0.87
Huesca	13	35.81	0.47	0.36	6	5.54	0.22	1.08
JaÃ©n	52	89.37	0.65	0.58	8	14.51	0.10	0.55
LeÃ³n	94	82.17	1.61	1.14	15	13.80	0.24	1.09
Lleida	23	60.99	0.44	0.38	4	9.55	0.08	0.42
Lugo	74	62.33	1.77	1.19	9	10.47	0.20	0.86
Madrid	536	783.19	0.72	0.68	108	138.01	0.13	0.78
MÃ¡laga	148	205.13	0.78	0.72	36	32.29	0.18	1.11
Melilla	3	8.06	0.31	0.37	0	1.20	0.00	0.00
Murcia	127	172.08	0.72	0.74	16	27.23	0.09	0.59
Navarra	99	89.55	1.29	1.11	31	14.37	0.40	2.16
Ourense	50	59.46	1.28	0.84	9	10.28	0.21	0.88
Palencia	37	28.61	1.78	1.29	6	4.64	0.28	1.29
Palmas, Las	288	131.53	2.21	2.19	27	19.69	0.21	1.37
Pontevedra	187	136.54	1.65	1.37	38	23.64	0.31	1.61
Rioja, La	57	46.02	1.49	1.24	7	7.32	0.18	0.96
Salamanca	56	56.78	1.35	0.99	2	9.58	0.05	0.21
S. C. Tenerife	154	128.37	1.30	1.20	18	20.07	0.15	0.90
Segovia	16	24.92	0.82	0.64	2	3.93	0.10	0.51
Sevilla	206	231.87	0.90	0.89	39	38.43	0.16	1.01
Soria	7	16.00	0.61	0.44	0	2.50	0.00	0.00
Tarragona	85	106.77	0.90	0.80	15	16.53	0.16	0.91
Teruel	21	23.77	1.19	0.88	0	3.61	0.00	0.00
Toledo	42	89.55	0.51	0.47	4	13.89	0.05	0.29
Valencia	270	337.75	0.89	0.80	46	56.01	0.15	0.82
Valladolid	58	79.60	0.91	0.73	5	13.03	0.08	0.38
Zamora	35	35.09	1.51	1.00	4	5.70	0.17	0.70
Zaragoza	99	138.84	0.86	0.71	17	23.20	0.14	0.73

1

## Data Availability

Declared none.
